# Causes, consequences, and perspectives in the variations of intestinal density of colonization of multidrug-resistant enterobacteria

**DOI:** 10.3389/fmicb.2013.00129

**Published:** 2013-05-28

**Authors:** Etienne Ruppé, Antoine Andremont

**Affiliations:** Laboratoire de Bactériologie, AP-HP, Hôpitaux Paris Nord Val de Seinesite Bichat-Claude Bernard, Paris, France

**Keywords:** carriage, concentration, extended-spectrum beta-lactamases, microbiota, antibiotics

## Abstract

The intestinal microbiota is a complex environment that hosts 10^13^ to 10^14^ bacteria. Among these bacteria stand multidrug-resistant enterobacteria (MDRE), which intestinal densities can substantially vary, especially according to antibiotic exposure. The intestinal density of MDRE and their relative abundance (i.e., the proportion between the density of MDRE and the density of total enterobacteria) could play a major role in the infection process or patient-to-patient transmission. This review discusses the recent advances in understanding (i) what causes variations in the density or relative abundance of intestinal colonization, (ii) what are the clinical consequences of these variations, and (iii) what are the perspectives for maintaining these markers at low levels.

## INTRODUCTION

Microbiota refers to the microorganisms (bacteria, archaea, and yeasts) that colonize the epithelial and mucosal surfaces that are exposed to the outside environment: the skin, oropharynx, vagina, and intestine. The microbiota that inhabit the intestines, from the duodenum to the rectum, are defined as intestinal microbiota. Its composition and density vary along the digestive tract, the colon hosting much denser and more complex microbiota than the upstream regions of the intestine (Stearns et al., 2011). The composition of this intestinal microbiota is reflected, albeit incompletely, by that of the feces (Durban et al., 2011; Stearns et al., 2011). In humans, the intestinal microbiota is composed of 10^13^ to 10^14^ microbial cells, which exceeds the total number of cells that compose the host body (Whitman et al., 1998). Most intestinal bacteria are strict anaerobic bacteria which are infrequently human pathogens. Conversely, enterobacteria make up a small proportion of intestinal microbiota (1/10^4^) but are major human pathogens whose steadily increasing antibiotic resistance poses a significant health threat.

Increased knowledge of the composition of intestinal microbiota and the effect of antibiotics became evident in the last decade because of major technological improvements in DNA sequencing (next-generation sequencing; Qin et al., 2010; Dethlefsen and Relman, 2011; Taur et al., 2012). Antibiotics have a twofold effect on the intestinal microbiota: (i) loss of bacterial diversity and (ii) overgrowth of resistant bacteria in the niches left by susceptible bacteria. Thus, antibiotic treatment can increase the density and relative abundance of resistant bacteria in the intestinal microbiota. Indeed, a high density and/or high relative abundance of resistant bacteria may be linked to a higher risk of infection or patient-to-patient transmission. In this review, we address this question with a special emphasis on multidrug-resistant enterobacteria (MDRE). Finally, we will review the recent advances in strategies to keep MDRE at low intestinal densities.

## DESCRIPTION OF HUMAN INTESTINAL MICROBIOTA

### COMPOSITION OF THE INTESTINAL MICROBIOTA

The human intestinal microbiota is composed of nine bacterial divisions, which is far less than the number observed in soils (at least 20 divisions; Ley et al., 2006). Most gut colonizers do not grow outside the gut and are transmitted via direct person-to-person contacts. Children inherit their microbiota from their mothers (Dominguez-Bello et al., 2010), and intestinal microbiota is highly similar between identical twins (Turnbaugh et al., 2010). The MetaHIT project^1^, a vast metagenomics study aimed at establishing an exhaustive catalog of the genes present in the intestinal microbiota, found that from the feces of 124 individuals, 18 species were found to be common to all individuals, and 57 were present in ≥90% individuals (Qin et al., 2010). Among those 57 species, the most abundant were from the phyla Bacteroidetes and Firmicutes, yet up to 2000-fold variation in relative species abundance was observed among individuals. Fecal enterobacteria, which mostly comprise *Escherichia coli*, are subdominant bacteria, making up to 10^8^ to 10^9^ colony-forming units (CFU) per gram of feces.

### THE BARRIER EFFECT REGULATES THE DENSITIES OF COLONIZATION AMONG THE BACTERIAL POPULATIONS OF THE INTESTINAL MICROBIOTA

The intestinal microbiota is mostly composed of prokaryotes, even if eukaryotes, such as fungi, are also present. However, the intestinal microbiota plays a role of an organ in humans and provides various benefits to its host. One benefit is the barrier effect or colonization resistance, which refers to the ability of intestinal microbiota to prevent sustainable colonization by exogenous bacteria (Ducluzeau et al., 1977; Vollaard and Clasener, 1994). Most ingested bacteria only transit through the digestive tube and do not colonize the patient for a significant period of time (Buck and Cooke, 1969; Cooke et al., 1972). The barrier effect mainly relies on the fact that the endogenous intestinal microbiota leaves very few niches (available nutrients and attachment sites) for use by exogenous bacteria (Vollaard and Clasener, 1994). The intestinal microbiota is also involved in the maintenance of intestinal epithelial homeostasis (Rakoff-Nahoum et al., 2004) and promotes angiogenesis (Stappenbeck et al., 2002). Moreover, a link between the intestinal microbiota and metabolic disorders has been proposed; in particular, low species diversity in the intestinal microbiota has been linked to medical conditions, such as obesity, inflammatory bowel diseases, atopy, and diabetes (Ley et al., 2005; Turnbaugh et al., 2006; Round and Mazmanian, 2009; Qin et al., 2012).

### THE INTESTINAL MICROBIOTA IS A VAST RESERVOIR FOR RESISTANCE GENES

Next-generation sequencing has not yet changed the way antibiotic resistance is investigated, perhaps because the resistance genes that may be harbored by subdominant bacteria remain inaccessible to the current sequencing methods (Lagier et al., 2012b). However, the intestinal tract is a major reservoir for antibiotic-resistant bacteria, including naturally resistant bacteria and those with acquired resistance-conferring genes carried on mobile genetic elements such as plasmids, conjugative transposons or integrative and conjugative elements (ICEs; Sommer et al., 2009). The diversity of the resistance genes among intestinal bacteria (i.e., the intestinal resistome) cannot be effectively assessed by conventional methods based upon culture on antibiotic-supplemented agar media because most intestinal bacteria cannot be cultured using conventional methods. The use of various culture media, atmospheric conditions and mass-spectrometry identification (“culturomics”) allowed the establishment of an inventory of bacterial species, including many new intestinal microbiota species, in numbers even greater than those discovered using pyrosequencing (Lagier et al., 2012a). Thus far, this technique has not addressed the question of global antibiotic resistance of the microbiota.

Currently, the best description of the diversity of resistance genes present has been obtained using culture-independent methods (Sommer et al., 2009). Sommer et al. (2009) cloned the metagenome of feces samples into susceptible *E. coli* and plated it on agar media supplemented with various antibiotics. When applied to an overnight aerobic culture of feces, 95% of the identified genes had >90% nucleic identity with sequences in GenBank^2^ found in pathogenic bacteria. Indeed, the genes identified in the aerobic fraction had been reported, repeatedly, to occur on mobile genetic elements [*bla*_CTX-M_, *bla*_TEM_, *aac(3)-II*, *aac(6′)-Ib*, *bla*_AmpC_] found in pathogenic bacteria, such as enterobacteria. Conversely, when applied to the feces metagenome with no previous aerobic culture, the average shared identity dropped to 60.7%. Thus, the intestinal resistome can be divided into (i) a “resident” resistome, composed of the resistance genes naturally present in permanent members of the intestinal microbiota, such as the beta-lactamase gene from *Bacteroides* sp., and (ii) a variable resistome, composed of exogenous genes present in transient bacteria or acquired by lateral transfer (Wellington et al., 2013). Enrichment of the variable resistome comes from ingestion of resistant bacteria through food (Ruimy et al., 2010) or fecal peril (Tangden et al., 2010). Although exogenous bacteria may not colonize because of the intestinal microbiota barrier effect (also called “resistance to colonization”), their resistance genes can be transferred to resident bacteria through horizontal gene transfer during transit (Duval-Iflah et al., 1980).

## MULTIDRUG-RESISTANT ENTEROBACTERIA: A FOCUS ON BETA-LACTAMS

Antibiotics have been extensively used since the 1950s with a parallel increase in the proportion of resistant bacteria (Clatworthy et al., 2007). Indeed, there are no more or no fewer bacteria since antibiotics were initiated; yet, there are more resistant bacteria. Between 1950 and 1980, the continuous discovery of new and more potent antibiotics has conferred to medicine a constant advantage over the rise of bacterial resistance. As long as new antibiotics were made available on a regular basis, resistance was not a real problem because clinicians always had drugs to which bacteria were susceptible to treat patients. Still, resistance never slowed down and benefited from extensive international exchanges to spread worldwide (MacPherson et al., 2009). Meanwhile, the pipeline of new, effective antibiotics has nearly ceased (Spellberg et al., 2004). The efficacy of beta-lactams, the most widely used antibiotic family worldwide, is now challenged by the spread of the so-called “bad bugs,” e.g., enterobacteria, *Pseudomonas aeruginosa* and *Acinetobacter baumannii* that produce wide-spectrum beta-lactamases (**Table 1**; Peterson, 2009). Until the early 2000s, such resistant bacteria were isolated quasi-exclusively in healthcare structures and did not affect community patients. Successful interventions to control their spread in healthcare structures have been developed and are now part of usual care (Lucet et al., 1999). Yet, the situation has dramatically changed with the emergence and dissemination in the community of enterobacteria that produce CTX-M – type extended-spectrum beta-lactamases (ESBL; Pitout and Laupland, 2008). Occurrence of CTX-M beta-lactamases is especially prominent in developing countries, maybe because of uncontrolled antibiotic consumption and suboptimal hygienic living conditions (Ruppe et al., 2009; Woerther et al., 2011).

**Table 1:** Main acquired beta-lactamases produced by Gram-negative bacilli from the intestinal microbiota.

Name of beta-lactamase	Type of enzyme	Beta-lactam spectrum of hydrolysis	First-line alternative drugs	Bacterial hosts	Presence in community	Prevalence
CTX-M gp 1	ESBL	PENI, CEPH, ATM	CBP, TGC, COL	Ent, Pyo, Acineto	Yes	Very high
CTX-M gp 2	ESBL	PENI, CEPH, ATM	CBP, TGC, COL	Ent, Pyo	Yes	Very high
CTX-M gp 25	ESBL	PENI, CEPH, ATM	CBP, TGC, COL	Ent	Yes	High
CTX-M gp 8	ESBL	PENI, CEPH, ATM	CBP, TGC, COL	Ent	Yes	High
CTX-M gp 9	ESBL	PENI, CEPH, ATM	CBP, TGC, COL	Ent	Yes	Very high
SHV-type ESBL	ESBL	PENI, CEPH, ATM	CBP, TGC, COL	Ent, Pyo, Acineto	Yes	Very high
TEM-type ESBL	ESBL	PENI, CEPH, ATM	CBP, TGC, COL	Ent, Pyo, Acineto	No	High
IMP gp 1 and 2	CP	PENI, P + I, CEPH, CBP	TGC, ATM, COL	Ent, Pyo, Acineto	No	Low
KPC	CP	PENI, CEPH, ATM, CBP	TGC, COL	Ent, Pyo	No	High
NDM-1	CP	PENI, P + I, CEPH, CBP	TGC, ATM, COL	Ent, Pyo, Vibrio	Yes	High
OXA-48	CP	PENI, P + I, CBP	CEPH, TGC, COL	Ent	Yes	High
VIM gp 1 and 2	CP	PENI, P + I, CEPH, CBP	TGC, ATM, COL	Ent, Pyo, Acineto	No	High
Cit-group AmpC (CMY-2)	AmpC	PENI, P + I, CEPH	C3G, TGC, CBP	Ent	Yes	High
Other plasmidic AmpC	AmpC	PENI, P + I, CEPH	C3G, TGC, CBP	Ent	No	Low

*ESBL, extended-spectrum beta-lactamase; PENI, penicillins; CEPH, cephalosporins; ATM, aztreonam; CBP, carbapenems; TGC, tigecycline; COL, colistin; Ent, *Enterobacteriaceae*; Pyo, *Pseudomonas aeruginosa*; Acineto, *Acinetobacter* baumannii; CP, carbapenemase; P + I, penicillin + class A beta-lactamase inhibitor; AmpC, cephalosporinase.*

Therapeutic options for patients infected by ESBL-producing enterobacteria (ESBL-E) remain limited to a few antibiotics, including carbapenems. Thus, the rise of ESBL-E fuels a cycle of increased carbapenem consumption. This cycle leads to the dissemination of carbapenem-resistant enterobacteria (CRE). Carbapenem resistance in enterobacteria occurs through either porin loss (Skurnik et al., 2010) or carbapenem-hydrolyzing enzyme (“carbapenemase”; Queenan and Bush, 2007; Nordmann et al., 2009; Kumarasamy et al., 2010; Poirel et al., 2012). There are two main concerns regarding CRE. First, carbapenemase are derivatives of class A, B, and D beta-lactamases, and some have been repeatedly recovered from patients with no recent history of hospitalization or travel abroad (Vaux et al., 2011) or in the community environment, such as in tap water in India (Walsh et al., 2011). The dissemination of CRE in the community would be exceedingly difficult to halt, as observed for CTX-M. Second, very few or no antibiotics have activity toward CRE, and infections caused by some of these bacteria are not treatable with our current armamentarium. The WHO has classified antibiotic resistance as one of the three major current threats to health^3^. More efficient control of ESBL-E should lead to the decreased use of carbapenems, which in turn, should slow down the dissemination of carbapenemases.

## ANTIBIOTICS AS A CAUSE OF VARIATIONS IN THE INTESTINAL DENSITY OF COLONIZATION OF RESISTANT BACTERIA

### QUANTITATIVE IMPACT: LOSS OF DIVERSITY

Antibiotic effects on the intestinal microbiota depend on (i) the colonic concentrations of the antibiotic (luminal and mucosal) and/or its active metabolites and (ii) the activity of these concentrations on the bacterial species present. The growth of susceptible bacteria will either be impeded (bacteriostatic effect), or they will be killed (bactericidal effect; Dethlefsen et al., 2008; Antonopoulos et al., 2009). Thus, the extent and persistence of the impact of antibiotics on the intestinal microbiota is highly drug-dependent (Nord et al., 1984; Taur et al., 2012). Even antibiotics of the same family and spectrum of activity can have a very different impact depending on their rate of intestinal excretion (Brautigam et al., 1988; Michea-Hamzehpour et al., 1988). Using next-generation sequencing, Dethlefsen and Relman (2011) precisely observed the fecal diversity of three healthy subjects during 300 days, during which they received 2 × 5-day courses of ciprofloxacin, at days 60 and 250. Ciprofloxacin caused a loss of diversity and a shift in community composition occurring within 3–4 days of drug initiation. This effect was somewhat surprising because most of the microbiota is composed of anaerobes that are weakly susceptible to ciprofloxacin (Nord and Edlund, 1989). However, concentrations of ciprofloxacin that accumulate in the colon during treatments (Fantin et al., 2009) are so high that they most likely overcame their minimal inhibitory concentrations. The perturbation created by antibiotic use took weeks to be resolved; furthermore, the composition of the intestinal microbiota remained altered from its initial state.

In newborns treated by a combination of ampicillin and gentamicin, the Actinobacteria and Firmicutes phyla, comprising bacteria with potential benefit (*Bifidobacterium* and *Lactobacillus*) were replaced by Proteobacteria, including Enterobacteriaceae (Fouhy et al., 2012). This effect could be of importance considering that *Proteobacteria* are enriched with mobile genetic elements, including antibiotic resistance encoding genes (Baquero et al., 2013). The increase of *Proteobacteria* was persistent after 8 weeks. Indeed, in patients undergoing allogeneic hematopoietic stem cell transplantation, metronidazole (an antibiotic with broad-spectrum activity against anaerobes) strongly reduced the diversity of the intestinal microbiota (Taur et al., 2012). In mice receiving a combination of amoxicillin, metronidazole, and bismuth, the composition of the intestinal microbiota was altered, but the perturbation was resolved within 2 weeks; in contrast, resolution took approximately 6 weeks for mice treated with cefoperazone, a wide-spectrum cephalosporin (Antonopoulos et al., 2009). The resilience of the composition of the intestinal microbiota is thus likely to be different according to the type of antibiotic given.

### QUALITATIVE IMPACT: LOSS OF THE BARRIER EFFECT

**FIGURE 1:**
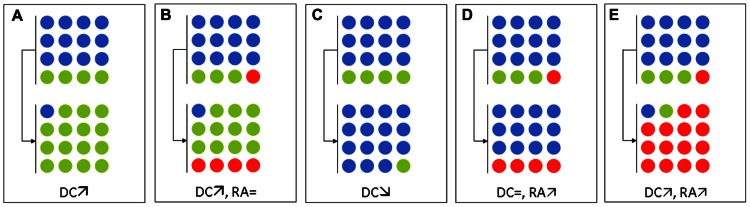
**Schematic representation of the effects of various antibiotic regimens on the intestinal microbiota with regard to multidrug-resistant enterobacteria (MDRE).** Blue, green, and red circles represent anaerobes, antibiotic-susceptible enterobacteria, and MDRE, respectively. **(A,B)** Antibiotics active against anaerobes (e.g., metronidazole, clindamycin, vancomycin) in an MDRE–non-carrier **(A)** and in an MDRE–carrier **(B)**. **(C,D)** Antibiotics with no activity against anaerobes, but are active against enterobacteria (e.g., fluoroquinolones, cefepime, co-trimoxazole) in an MDRE–non-carrier **(C)** and in a MDRE–carrier **(D)**. **(E)** Antibiotics with activities against both anaerobes and enterobacteria (e.g., ceftriaxone, co-amoxiclav) in an MDRE–carrier. DC, density of colonization of MDRE; RA, relative abundance of MDRE.

As described above, the barrier effect is mainly exerted by anaerobes (Ducluzeau et al., 1977; Vollaard and Clasener, 1994). Thus, antibiotics with high activity against anaerobes, such as clindamycin, potently affect the capacity of the microbiota to prevent colonization by exogenous microorganisms (van der Waaij et al., 1971). In the study by Donskey et al. (2000), 13 vancomycin-resistant enterococci (VRE)-carriers received antibiotics active against anaerobes, while 10 VRE-carriers received antibiotics poorly active against anaerobes . Strikingly, the average density of VRE (expressed in log CFU/g of feces) significantly increased (by 2.2 logs) in patients who received antibiotics that were active against anaerobes, whereas the average density decreased by 0.6 log in those patients receiving antibiotics with minimal activity against anaerobes. In a subsequent study, the same group has reported that the density of resistant Gram-negative bacilli also increased under exposure to antibiotics that were active against anaerobes (Bhalla et al., 2003). In the latter case, the resistant bacteria occupied niches that appeared to be left by anaerobes (**Figure 1**).

The barrier effect can also be studied by considering one species, such as *E. coli*. In healthy volunteers, the *E. coli* population is composed of a variable number of clones of different abundances: dominant and subdominant clones (Lidin-Janson et al., 1978). When these *E. coli* have different susceptibilities, antibiotic exposure will change their respective proportions and promote the growth of the resistant strains over that of the susceptible ones (**Figure 1**). This phenomenon has recently been reported for fluoroquinolones, which cause a sharp decrease in total counts of intestinal enterobacteria during treatments. The available niches can then be occupied by resistant enterobacteria that were initially present in low fecal concentrations (Fantin et al., 2009) or from a new acquisition (de Lastours et al., 2012). If the number of total enterobacteria remains unchanged, then the relative abundance of resistant enterobacteria increases (**Figure 1**).

## CONSEQUENCES OF INCREASED DENSITY OF COLONIZATION ON INFECTIONS

### ANTIBIOTICS INCREASE THE RISK OF INFECTIONS CAUSED BY RESISTANT ENTEROBACTERIA

Several studies have shown that patients with infections caused by resistant bacteria were more likely to have taken antibiotics recently (Ben-Ami et al., 2009). Indeed, the link between antibiotic exposure and antibiotic-resistant infection could lie in the intestinal microbiota, as antibiotics would allow the overgrowth of resistant bacteria (i.e., increase the density of resistant bacteria). Interestingly, the association between antibiotic use and infections caused by resistant bacteria is also found for antibiotics with little effect on anaerobes, e.g., co-trimoxazole or quinolones, suggesting that it is not only the overgrowth of resistant bacteria within niches left empty by anaerobes that increases the risk of infection by resistant bacteria, but more likely the augmentation of the relative abundance of resistant bacteria (i.e., the augmentation of the proportion of resistant bacteria) within specific niches (**Figure 1**). MDRE are extensively antibiotic-resistant and not only to beta-lactams, but also to many other antibiotics including among others fluoroquinolones, aminoglycosides, or co-trimoxazole (Pitout, 2009). Thus, MDRE can overgrow and increase the risk of their involvement in further infection under almost any antibiotic exposure.

### URINARY-TRACT INFECTIONS

Urinary-tract infections (UTIs) are the most common bacterial infections (Hooton, 2012) and are most often caused by enterobacteria, and especially *E. coli*. There is some evidence that in most cases, the infecting clone originates from the intestinal microbiota (Yamamoto et al., 1997), even if it cannot always be retrieved in the stool at the onset of the symptoms (Moreno et al., 2008). Specific strains of *E. coli* appear to have the ability (through virulence factors) to colonize the urethra and bladder, causing cystitis; others are able to further colonize the ureter to cause pyelonephritis (Plos et al., 1995). According to this “virulence theory,” some strains of *E. coli* could cause UTIs, even if they are subdominant in the intestines (low relative abundance). Alternatively, the relative abundance of the various clones of *E. coli* present in the feces may also play a key role in the pathogenesis of UTIs, in that the dominant clone of *E. coli* would have the maximum likelihood to colonize the urinary tract (Moreno et al., 2008). In 42 women with cystitis caused by *E. coli*, Moreno et al. (2008) compared the UTI-causing strain to 30 randomly picked colonies from concomitant stool samples. In 90% of the women, the urine clones were found in the feces. In 71% of the women, the urine clone that was present in the feces was also the dominant fecal clone. Urine clones mostly belonged to B2 and D phylogroups and had an increased content of urovirulence factors. For one given fecal clone, predictors of infection were that the urine clone belonged to the B2 phylogroup and was dominant. Thus, the findings of this study reconciled the dominance and the virulence theories.

### BACTEREMIA

Translocation is defined as the passage of viable indigenous bacteria from the gastrointestinal tract to the mesenteric lymph nodes (Berg and Garlington, 1979). This passage is the first step for bacteria to settle in other locations, such as the bloodstream, or cause secondary forms of infections, such as in the cardiac valves. Bacterial translocation can be part of normal physiological processes in healthy subjects, but to a limited extent and without deleterious consequences. By contrast, sustained translocation is observed in subjects with specific deficiencies, such as neutropenia, starvation, or hemorrhagic shock and then leads to severe septic consequences (Tancrede and Andremont, 1985; Youssef et al., 1998). A key point determining bacterial translocation is the intestinal density: the translocating bacteria are mostly dominant within the intestinal microbiota (Youssef et al., 1998; Taur et al., 2012). Furthermore, translocation of enterobacteria has been reported in immunocompetent mice exposed to penicillin, clindamycin, and metronidazole (Berg, 1981). In the same report, the measured intestinal density of enterobacteria increased by 3–5 logs. Thus, antibiotics that increase the density of resistant bacteria would increase the risk of their translocation.

### PATIENT-TO-PATIENT CROSS-TRANSMISSION

In the above-mentioned study from Donskey et al. (2000), the surrounding environment of 10 VRE-carrying patients was investigated for the presence of VRE. Environmental samples from 21 patients were analyzed and compared according to the intestinal density of VRE. Strikingly, when the density was <4 logs CFU/g of stool, VRE were found in the patient’s environment only in one out of nine sample sets (11%). Conversely, when density was ≥4 logs CFU/g of stool, VRE were found in 10 of 12 sets (83%). Other studies have shown that patients can acquire resistant bacteria from a former occupant of the room through the persistence of the bacteria on environmental surfaces (Datta et al., 2011). Although no study has shown that cross-transmission occurs less often when the density of resistant bacteria was low, the results from Donskey et al. (2000) support this notion. To date, no data are available for MDRE.

## PERSPECTIVES: HOW TO DECREASE THE DENSITIES OF COLONIZATION OF RESISTANT BACTERIA (Table 2; Figure 2)

### SELECTIVE DIGESTIVE DECONTAMINATION

**Table 2:** Options toward the modulation of the density and relative abundance of multidrug-resistant enterobacteria (MDRE) in the intestinal microbiota.

Name	Rationale	Effect on the IM	Advantages	Limitations	Phase	Reference
Selective digestive decontamination (SDD)	• Killing resistant bacteria	Loss of bacteria susceptible to the SDD regimen	• Simple	• Emergence of resistance to SDD agents • Causes damages to intestinal microbiota	In use	de Smet et al. (2011), Overdevest et al. (2011), Saidel-Odes et al. (2012)
Antibiotic colonic inactivation (ACI)	• Inactivating residues of antibiotics in the colon	None	• No effect on the intestinal microbiota	• Incomplete inactivation in the colon	Proof of concept	Tarkkanen et al. (2009), Khoder et al. (2010)
Fecal microbiota transplantation (FMT)	• Restoration of a new intestinal microbiota by allo- or auto-transplantation	Restoration after antibiotics	• Restore a full, healthy intestinal microbiota with barrier effect • Prevent *C. difficile* infection	• Acceptance • Transmission of pathogens	Used occasionally	Gough et al. (2011), Ubeda et al. (2013), van Nood et al. (2013)
Antibiotic stewardship programs (ASPs)	• Prioritizing antibiotics with minimal effects on the intestinal microbiota • Avoiding inappropriate prescriptions	The least effect as possible	• Optimized infection management for patients	• Necessity of a close collaboration between trained clinicians and biologists • In some cases, the use of wide-spectrum antibiotics cannot be avoided	In use	MacDougall and Polk (2005), Davey et al. (2005)

**FIGURE 2:**
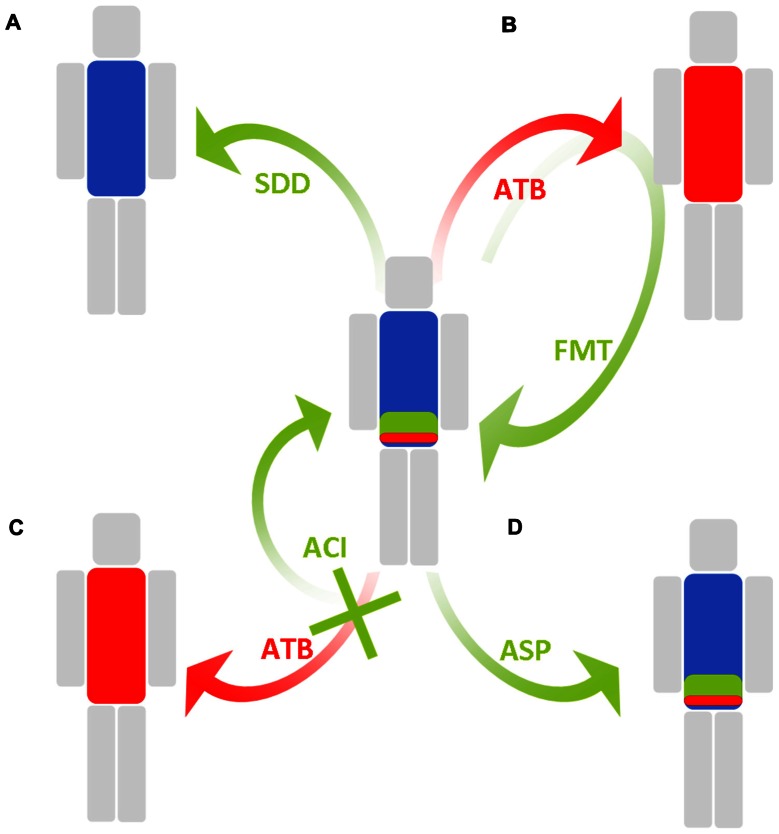
**Schematic representation of solutions employed to maintain a low density of colonization of multidrug-resistant enterobacteria in the intestinal microbiota**. Blue, green, and red, respectively, represent anaerobes, antibiotic-susceptible enterobacteria, and MDRE. **(A)** Selective digestive decontamination (SDD) eradicates all enterobacteria, including MDRE. **(B)** Fecal microbiota transplantation (FMT) reinstalls the original intestinal microbiota or an intestinal microbiota from a healthy donor after antibiotic-induced perturbations. **(C)** Antibiotic colonic inactivation (ACI) inactivates antibiotics and residues when they reach the colon. **(D)** Antibiotic stewardship program (ASP) favors antibiotics with minimal impact on the intestinal microbiota.

Selective digestive decontamination (SDD) aims to eliminate MDRE from the intestinal lumen and prevent further infection and dissemination. In SDD, the patient receives orally and/or parenterally administered wide-spectrum antibiotics for a short time, with the aim of eradicating most potentially pathogenic bacteria and/or the resistant bacteria from the intestinal microbiota, while sparing anaerobes as much as possible. SDD has mostly been used to eliminate all enterobacteria in patients at risk of infections, such as in onco-hematology and intensive care ones. In 1985, SDD was successfully employed in France for the control of a major outbreak involving MDRE (Brun-Buisson et al., 1989). The SDD regimen comprised oral neomycin, colistin, and nalidixic acid. The rate of MDRE infections decreased, yet the overall rate of nosocomial infections did not.

More recently, two decontamination regimens have been compared: selective oropharyngeal decontamination (SOD: an oropharyngeal topical administration of tobramycin, colistin, and amphotericin B, an antifungal agent) and SDD (SOD + intravenous cefotaxime), each given for 4 days (de Smet et al., 2011). SDD and SOD caused a slight reduction in mortality compared to the control group. Unexpectedly, SDD-receiving patients carried fewer enterobacteria resistant to ciprofloxacin, ceftazidime, or aminoglycosides than the standard care group. In a later study, the authors showed that when no Gram-negative bacilli were found in the intestinal microbiota, the rate of Gram-negative bacilli bacteremia decreased threefold (Oostdijk et al., 2011). However, if patients continued to be colonized, the rate of bacteremia was not different from that of patients receiving standard care (Oostdijk et al., 2011).

A recently published *post hoc* analysis focused on a subgroup of 507 patients with detectable Gram-negative bacilli in their intestinal microbiota prior to SDD (Oostdijk et al., 2012). As expected, the eradication rates for enterobacteria differed according to the resistance pattern. The difference was statistically significant for aminoglycoside resistance (62 vs. 81% in the standard care group; *p* < 0.05), but only a trend was evidenced for third-generation cephalosporins resistance (73 vs. 80% in the standard care group; *p* = 0.053). The study did not mention whether colistin-resistant bacteria were eradicated or not. Indeed, SDD raises the issue of using a last-resort antibiotic, colistin, with the risk of selecting MDRE in the microbiota which would also be resistant to the drug (Kumarasamy et al., 2010). So far, SDD has mainly been studied in countries with low MDRE prevalence (de Smet et al., 2011; Overdevest et al., 2011) and this risk could be much higher in countries where it is high.

A study in Israel, a country with a high prevalence of bacteria that produce the *Klebsiella pneumoniae* carbapenemase (KPC)-type carbapenemase, has tested a 7-day colistin + gentamicin SDD regimen in patients colonized with such bacteria (Saidel-Odes et al., 2012). The results showed that at day 7 (at the end of SDD), KPC-producing bacteria were no longer detectable in the feces of 61% of patients vs. 16% in placebo. Nevertheless, when SDD was discontinued, KPC-producing bacteria could be detected again in some formerly negative patients, suggesting that the concentrations of these bacteria were below the limit of culture detection while SDD was being applied. Nevertheless, keeping MDRE at low intestinal concentrations in the absence of total eradication may be sufficient to prevent further infections or cross-transmission. Interestingly, no colistin or gentamicin resistance emerged among the recovered KPC-producing bacteria.

### INACTIVATION OF ANTIBIOTICS IN THE INTESTINE

Instead of killing resistant bacteria, another approach would be to prevent their overgrowth by inactivating the antibiotics in the intestine during treatments. Orally administered antibiotics are primarily absorbed in the proximal jejunum, yet a fraction reaches the colon, where the density of bacteria is maximal. Parenterally administered antibiotics are filtered by the liver, and a fraction is excreted through the gall bladder into the jejunum and then reaches the colon. Thus, the concept of designing drugs or beta-lactamases capable of inactivating antibiotics in the colon, but not earlier, has arisen.

The protective effect of a recombinant beta-lactamase, P1A, has been evaluated in 34 human volunteers taking ampicillin (Tarkkanen et al., 2009). In the ampicillin group without P1A, a decrease of *Bifidobacterium*, *Streptococcus*, *Lactobacillus*, and an increase of *E. coli* and yeasts, were noted in the intestinal microbiota. Conversely, in the ampicillin + P1A group, no significant changes in the composition of the intestinal microbiota could be observed. Furthermore, the relative abundance of ampicillin-resistant *E. coli* increased from 2.1% at baseline to >72.7% at day 5 under ampicillin only, while it remained under 10% in the ampicillin + P1A group. In addition to target-specific inactivation strategies, adsorbents, such as colonic delivery of activated charcoal, were shown to be efficient in trapping ciprofloxacin in a rat model (Khoder et al., 2010). Further clinical studies are expected to assess the efficacy of such a strategy.

### PROBIOTICS AND FECAL MICROBIOTA TRANSPLANTATION

Probiotics are defined as live microorganisms that may confer a health benefit to their host. The most used probiotics are lactic acid bacteria, *Bifidobacteria*, *E. coli* Nissle 1917 or *Saccharomyces boulardii*, a yeast. It is unknown if these probiotics could exert a barrier effect for resistant bacteria. A study from New Zealand competed *E. coli* Nissle with a fluoroquinolone-resistant *E. coli* in elderly residents, and the results showed that there was eventually no difference in terms of carriage of the fluoroquinolone-resistant *E. coli* between the Nissle group and the placebo group (Tannock et al., 2011). Another growing concern about probiotics is that there remains little evidence that the massive ingestion of one single species can restore all the intestinal microbiota at a significant extent. Another potential way to restore microbiota is a fecal microbiota transplantation (FMT), which refers to the process of instilling a liquid suspension of stool from a healthy donor into the gastrointestinal tract of a patient to restore the intestinal microbiota immediately after any perturbation, such as that caused by antibiotics.

When mice with intestinal microbiota affected by antibiotics, were caged with mice without previous antibiotic exposure, a faster restoration of the intestinal microbiota was observed. It was suggested that this was due to the transfer of a normal microbiota to the antibiotic treated mice, likely through coprophagy (Antonopoulos et al., 2009). In humans, FMT has demonstrated high efficacy in patients with recurrent *Clostridium difficile* infections (Gough et al., 2011; van Nood et al., 2013). The main limitation of FMT is its obvious repellence, which could be overcome by rectal instillations instead of oral routes (Bakken, 2009). Another caveat is that the fecal samples administered could include undetected pathogens. This caveat could be overcome by auto-banking (Tosh and McDonald, 2012) or by using preparation containing a cocktail of defined strains (Hamilton et al., 2012). This has recently been done to lower the density of VRE in mice (Ubeda et al., 2013) and of MDRE in chickens (Nuotio et al., 2013). However, these approaches have not been used in humans so far. If data support the efficacy of FMT in resolving *C. difficile* infections, studies assessing its efficacy in the context of outbreaks of MDRE to lower the risk of their transmissions and infections are warranted.

### ANTIBIOTIC STEWARDSHIP PROGRAMS

Another strategy for combating antibiotic-induced perturbations and to keep MDRE at low densities is to improve the use of antibiotics through antibiotic stewardship programs (ASPs; MacDougall and Polk, 2005). Indeed, these programs can be beneficial to the intestinal microbiota at three levels: (i) avoid prescriptions when antibiotics are not justified (Willemsen et al., 2010), (ii) scale down from the use of empirical wide-spectrum antibiotics to the narrowest spectrum possible, guided by antibiotic-susceptibility tests (Cosgrove et al., 2007), and (iii) choose the antibiotic with the lowest impact on the intestinal microbiota whenever possible (Lesprit and Brun-Buisson, 2008). In a Cochrane-based review, ASPs have been showed to decrease the overall antibiotic resistance, as well as *C. difficile* infection, suggesting their role in intestinal microbiota preservation (Davey et al., 2005). To reduce the use of wide-spectrum antibiotics further, new rapid diagnostic tests that identify resistance traits in strains in clinical samples or feces are being developed and have attracted interest from clinicians (Cuzon et al., 2012).

## CONCLUSION

The intestinal microbiota has to be considered an organ that is mistreated with every antibiotic administration. The intestinal microbiota provides several benefits to its hosts, including colonization resistance. When it is disrupted, resistant bacteria overgrow in the empty niches. Although few data are available to date, it appears as though high densities of MDRE may increase the risk of further infections and transmissions between patients. Indeed, controlling levels of MDRE may be a key point in terms of care in the next few years; further studies in this regard are expected. In this perspective, simple methods to measure these quantitative parameters, such as qPCR instead of serial dilutions, are promising (Lerner et al., 2013).

## Conflict of Interest Statement

Etienne Ruppé: no conflict. Antoine Andremont is scientific adviser for the DaVolterra Company (www.davolterra.com) within the frame of the French law for innovation and research.

## Footnotes

1 www.metahit.eu

2 http://www.ncbi.nlm.nih.gov

3 http://www.who.int/topics/drug_resistance/en
